# The changing face of psychiatric training in the UK

**DOI:** 10.4103/0019-5545.58897

**Published:** 2010

**Authors:** M. Afzal Javed, M. A. Ramji, Robert Jackson

**Affiliations:** The Medical Center, 2 Manor Court Avenue, Nuneaton, CV11 5HX, UK; 1 ST2 in Psychiatry, London, SW1X 8PG, UK; 2 The Royal College of Psychiatrists, 17 Belgrave Square, London, SW1X 8PG, UK

**Keywords:** Psychiatry, medical education, post-graduate training

## Abstract

With the introduction of many reforms and changes in medical training in the UK, postgraduate training has undergone significant transformation. The establishment of Postgraduate Medical Education and Training Board (PMETB), Modernizing Medical Careers (MMC), new recruitment processes and changes in the curriculum and examination structure are all having a major impact on the future training and teaching programs in psychiatry in the UK. Entry into psychiatry is becoming increasingly competitive and progression in career is now competency based in addition to the examination requirements subject to an annual review and regular appraisal. A structured portfolio is also vital in order to present evidence of competencies and ensure smooth progression through the training grades. This paper gives a general outline of these changes and describes the new training and examination requirements of the new system in place in the Psychiatric training in United Kingdom.

## INTRODUCTION

The importance of postgraduate teaching and training in forming the knowledge base for future medical specialists is widely acknowledged. It is in this perspective that extensive changes are being proposed in the future higher medical teaching and training programs. The speciality of Psychiatry is also becoming more responsive to these changes, and keeping in view the significant development over recent years with expansion of knowledge in molecular biology, neurobiology, genetics, cognitive neurosciences, neuro-imaging, psychopharmacology and other related fields contributing to the growth of psychiatry, a number of new methods of teaching and training are being tried in many countries.[1] With an increase in the incidence and prevalence of mental disorders, it is likely that medical professionals will have to deal with an increased number of patients in their practices and this will therefore require more experienced and trained doctors at hospitals as well as at community levels.[2,3] The needs for having more psychiatrists and trained professionals therefore becomes inevitable as these doctors will not only play a pivotal role in reducing the burden of mental disorders but also being imparters of knowledge and skills to trainees, students and other multi-disciplinary staff; they will increase the repertoire of knowledge amongst a rapidly evolving speciality. Similarly an increased interest among newly qualified medical graduates to choose psychiatry as their future career makes it even more important for comprehensive reviews in the current training programs[4] and certainly requires continuing updates in initial and continual training programs.

In the UK, the Royal College of Psychiatrists have traditionally managed the specialist training in psychiatry and has been supervising the postgraduate training programs along with organizing the speciality examinations for many years that lead to the award of College Membership (MRCPsych).[5] Previously, the membership examination was comprised of two parts consisting of written and clinical examinations in each part, and the College was responsible for setting the curriculum and standards for these examinations. The system has however been revised with the recent transformation of postgraduate medical education across all specialties in the UK, and is now mainly driven by recent legislation and the current trends in service and healthcare delivery.[6] The Postgraduate Medical Education and Training Board (PMETB), an independent body directly accountable to Parliament, has taken over the responsibilities of the Specialist Training Authority of the Medical Royal Colleges and is now responsible for all aspects of postgraduate medical education, training and assessment.[7,8] Modernizing Medical Careers (MMC),[9] another initiative of the Department of Health, has also set out a career structure in which doctors are appointed to training programs with the original aim of streamlining the selection of trainees into training grades. It was initially proposed that the trainees would be awarded a national training number (NTN) and that they would progress through a ‘run-through’ program leading to the completion of training as long as they continue meeting defined competencies. This would make them eligible for award of certificate of completion of training (CCT) and entry onto the Specialist Register of the General Medical Council and hence they would be able to apply for the elusive consultant grades.

Following these changes, specialist training in psychiatry has witnessed significant reviews that are consistent with the new national trends and directions of PMETB and Department of Health initiatives.[10,11] Although some difficulties have been encountered in the implementation of the new system, it is however, becoming clear that these broad changes will remain in place and the focus will continue to be on a competency-based curriculum with key emphasis on assessments as reflected by the performance of the trainees while undertaking the training program.[12,13] However, as we explain later, there have been some changes in the way trainees progress from basic to advanced training and in the way recruitment is organized.

### Entry into psychiatric speciality training in the UK

August 2005 saw the introduction of the two-year foundation program[14] for new medical graduates to replace the one-year pre-registration House Officer role and the unified specialist training or ‘run-through’ program was introduced in 2007 for training in the various specialties. The structural reforms under the umbrella of MMC were designed to give doctors advancement in their career path based on attainment of competencies rather than based on time spent in a particular role, with the aim of improving patient safety through improved supervision and assessment against explicit criteria, which are set out in the curricula for each speciality.

Following graduation from medical school, junior doctors are currently required to undertake a two-year foundation program of six four-month posts. Foundation year 1 (F1) must consist of a minimum of four months of medicine and four months of surgery. Many other specialties can be chosen to make up the remainder of the foundation program. The common psychiatric specialties offered at foundation level are old age psychiatry, general adult psychiatry and child and adolescent psychiatry. Those keen for a career in psychiatry can opt to do one four-month post in F1 and a further four-month post in F2, although the aim of the foundation program is to equip junior doctors with a broad range of experiences and therefore they are encouraged to sample a wide range of specialties to broaden the experience before entering specialist training in their chosen field.[15]

### Progression in psychiatry: Career structure

Following F2, prospective trainees could apply for a specialist psychiatric training program that extends to a normal period of six year. The introduction of the new MMC career structure and a national online application system Medical Training application system (MTAS) brought the new system in place in 2007. Those entering from F2 were required to apply directly into speciality registrar year 1 (ST1), and those existing SHO trainees already on training rotations were asked to apply into the new system at the level most suitable for them against published specifications. In 2007, doctors had the possibility of applying to up to four specialties in one deanery, or one speciality in four different deaneries. There were two options for 2007 entrants: Entry into a ‘run-through’ grade which would mean issuing of a NTN through to completion of training at ST6, or entry into a ‘fixed-term speciality training appointment’ (FTSTA), which is a one-year post made up of three four-month posts or two six-month posts with educational approval. There would then be the possibility of entry into a further training grade the following year through open competition.

**Figure 1:**
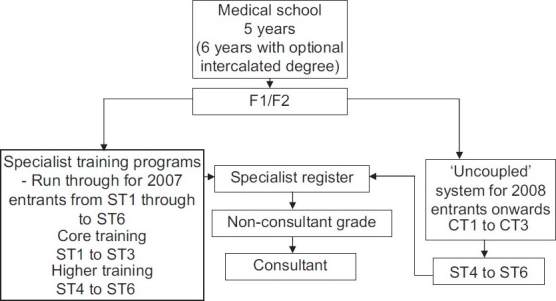
Programme structure

Unfortunately a number of difficulties emerged in the 2007 recruitment process and following the criticisms and the Tooke report which followed,[16] another major change took place of ‘uncoupling’ of certain specialties including psychiatry, medicine and surgery. It was decided that the 2007 entrants into a ST ‘run-through’ post were to have their training honored as continuous through to ST6 level. However those appointed to FTSTA posts in 2007 and all 2008 and onwards entrants would enter an ‘uncoupled’ system. This essentially means splitting of training to core training (CT) during the first three years of speciality training followed by higher speciality training during years four to six. Therefore, 2008 entrants to Core Training 1, 2 or 3 (which replaces ST 1, 2 and 3) would have to re-apply and compete for a higher specialist training post at the transition from CT3 to ST4. The entire structure as it now stands is summarized in Figure 1.

### Core training

The first three years of psychiatric training known as core training involves a minimum of 12 months experience in general adult psychiatry, with experience in child and adolescent psychiatry, old age psychiatry and psychotherapy in addition. Trainees must then choose their sub-speciality in which they will eventually be awarded their CCT. The College's examinations leading to the award of the MRCPsych must be passed during this time as a requirement for progression to ST4.

### Higher training

**Figure 2:**
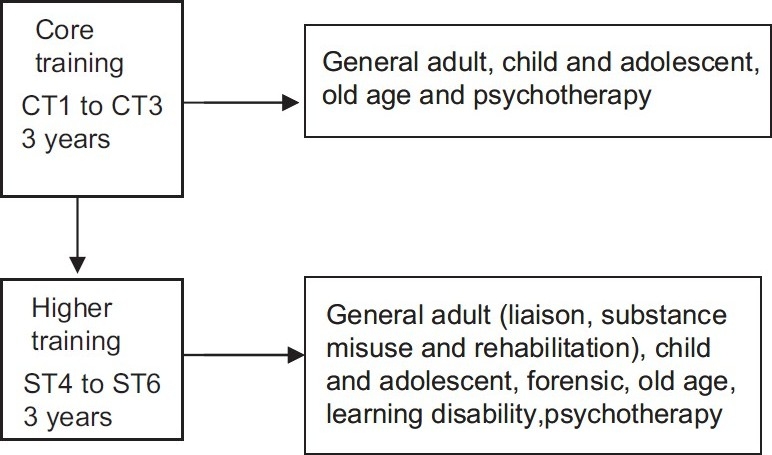
Higher speciality training

The higher speciality training now comprises six psychiatric specialties available for ST4 to ST6, namely general adult psychiatry (with three sub-specialties within this speciality: Liaison, substance misuse and rehabilitation), child and adolescent psychiatry, forensic psychiatry, old age, learning disability and psychotherapy [Figure 2]. It is also important to note that not all of the various specialties are available in each deanery and therefore trainees that have a strong interest in a particular sub-speciality need to be prepared to move regions during their training to pursue their specific choices.

### Progression through training period and required competencies

The new curriculum has placed greater emphasis on ‘Workplace-Based Assessments’ (WPBA).[17,18] There has been a lot of evidence for the WPBAs in assessing the competencies.[19–21] They have thus emerged as a compulsory element that all trainees must complete within specified time frames, which form part of the criteria to be able to progress through the training. The same WPBAs are used throughout the entire training program; however the standards expected for successful completion depend on the level of the trainee. The Royal College of Psychiatrists have outlined that the underlying principles of WPBAs must focus on performance, that is the work actually completed in the work place and these assessments must be evidence based and triangulated whereby the assessments should ideally be completed by different assessors on different occasions using different methods during each year of training.[22] The recording of these WPBAs is currently on paper as well as online via a dedicated website, but it is anticipated the move will be entirely towards electronic record keeping in the near future.

In order to progress through each year, each trainee is subject to an ‘Annual Review of Competence Progression’ (ARCP). This is a formal process where a panel reviews the evidence of progress to enable the trainee, postgraduate dean and employer to document that the competencies required have been gained at an appropriate rate. This replaces the ‘review of in-training assessments’ (RITA), which will continue to run for existing specialist registrars. The ARCP panel considers the adequacy of the evidence provided, and as long as evidence is presented, is able to make a judgment about a trainee's suitability to progress through training or make a judgment about whether a trainee is suitable for completion of training and therefore eligible for a CCT.

Workplace-based assessments have a formative function for effective review and appraisal providing a basis for feedback and educational planning. They also contribute towards the ARCP providing written evidence of attainment of competencies and therefore also play a summative role. There are eight WPBAs available to trainees.[22]

#### Assessment of clinical expertise

A trainer observes the assessment of a new patient undertaken by a trainee including a full history, mental state examination, diagnosis and management plan.

#### Mini-assessed clinical encounter

A trainee is observed for part of a patient interaction such as part of history taking or negotiating a treatment plan.

#### Case-based discussion

One of two recent patient clinical encounters is discussed allowing demonstration of decision-making and clinical knowledge.

#### Case presentation

This is an assessment of a clinical presentation delivered by a trainee in a meeting setting involving assessment of presentation skills and interpretation of evidence available.

#### Journal club presentation

This is used to assess the presentation of a journal article including analysis and critique.

#### Directly-observed procedural skills

Of limited use in psychiatry but can be used to assess administration of ECT or other procedures.

#### Mini-peer assessment tool

This works on the same principles as the previous 360 degrees appraisal, allowing co-workers from a range of disciplines to assess the trainees' attitudes, behaviors and ability to work in teams, but is entirely web-based.

#### Assessment of teaching

This is a new tool used to assess teaching skills, whether this is by lecture, tutorial or small group based.

The following Table 1 gives details of the required WBAs needed during the training period.

**Table 1:** Details of the WBAs during the training period

Year 1	Year 2	Year 3
2 ACE	3 ACE	3 ACE
4 Mini ACE	4 Mini ACE	4 Mini ACE
2 CBD	2 CBD	2 CBD
2 Mini PAT	2 Mini PAT	2 Mini PAT

At present, there are no set required numbers for WPBAs for ST4 to ST6, but higher trainees are encouraged to complete one WPBA per month. New tools are being developed and piloted for ST4 to ST6 trainees and should be introduced later this year.

At first the requirements may seem daunting but if trainees are well organized and complete WPBAs at regular intervals throughout each year of training, the task is much smoother. The original website used to record the WPBAs had teething difficulties but was developed further and brought under the College's control with significant improvements.

### The new MRCPsych structure and entry requirements

The structure of the membership examinations for the Royal College of Psychiatrists have also changed with the new training program and is now based on new methods of assessment.[18,22,23] The most up to date and comprehensive entry requirements for each paper are given in Table 2.

**Table 2:** The most up to date and comprehensive entry requirements for each paper

Requirement	Paper 1	Paper 2	Paper 3	Clinical assessment of skills and competencies
Training period	Mandatory 12 months	Recommended 18-24 months	Recommended 18-30 months	Minimum 30 months

Table 3 gives the details of theoretical topics covered in different papers.

**Table 3:** Theoretical knowledge

Paper 1	Paper 2	Paper 3
General adult, History and mental state examination, Cognitive assessment, Neurological examination, Assessment, Etiology, Diagnosis, Classification, Basic psychopharmacology, Basic psychological, processes, Human psychological, development, Social psychology, Description and measurement, Basic psychological treatments Prevention of psychiatric disorder, Descriptive psychopathology, Dynamic psychopathology, History of psychiatry, Basic ethics and philosophy of psychiatry Stigma and culture	General adult, General principles of psychopharmacology (pharmacokinetics, pharmacodynamics) Psychotropic drugs, Adverse reactions, Evaluation of treatments, Neuropsychiatry (physiology, endocrinology, chemistry, anatomy, pathology), Genetics Statistics and research (basic) Epidemiology Advanced psychological processes and treatments	General adult, Research methods, Evidence-based practice, Statistics Critical appraisal, Clinical topics, Liaison, Forensic, Addiction, Child and adolescent psychotherapy, Learning disability, Rehabilitation, Old-age psychiatry

In general, trainees applying to sit any of the written papers must have completed 12 months whole time equivalent training in psychiatry (a 12-month post in General Adult Psychiatry or a 6-month post in Old Age Psychiatry and a 6-month post of General Adult Psychiatry OR a combination of 4-month posts in General Adult or Old Age Psychiatry but with a minimum of 6 months in General Adult Psychiatry not counting any experience as a foundation trainee).

In order to sit paper 2, trainees are recommended to have between 18 and 24 months whole time equivalent training in Psychiatry.

The requirements for paper 3 include a recommendation of between 18 and 24 months whole time equivalent experience in psychiatry.

Trainees wishing to sit the final clinical examination, CASC (Clinical Assessment of Skills and Competencies) must have successfully completed the three written papers and have spent at least 30 months whole time equivalent placements in psychiatry. The CASC comprises twelve timed stations of 12 minutes each, including two minutes preparation time. Clinical Assessment of Skills and Competency is designed to test competency in clinical skills[7] and this examination is generally similar to the format of an OSCE examination.

Full details can be found in the Eligibility Criteria and Regulations for MRCPsych Examinations 2009.[5]

## CONCLUSION

The introduction of MMC, a new curriculum and a new training structure in the UK has seen psychiatric training transform with emphasis on the importance of evidence-based competencies in the workplace in addition to examination achievements. The presence of a structured portfolio is vital for trainees in the new program in order to present their achievements and competencies in a concise manner to enable a smooth progression through the new training structure. The intention of those behind the changes was to modernize and clearly structure a well-established system with a view to producing psychiatrists with enhanced skills and improved ability. With time we will see whether these changes achieve the desired goals.

It is also worth mentioning about another change in the training program that has involved the users and carers in this process. Following guidance in the National Service Framework for Mental Health that recommends service users and carers to be involved in planning and evaluating training, the involvement of service users and carers in an educational role is another recent development.[24] In June 2005, the Royal College of Psychiatrists proposed that all psychiatric trainees must have training from service users or carers, a significant move from patients previously only being involved in a passive way, as the possessor of symptoms and signs as a subject to study. It is argued that service users have a unique understanding of their illness and are best placed to judge trainees on their empathy and communication skills and hence their views are increasingly being valued and sought during training and examination of medical students and doctors.[24,25]

The new system is however facing a number of problems. As a result of an absence of any pilot of the new structure or the online application form, the 2007 entry into speciality training had to be modified several times during the actual application and interview period itself. The Medical Training Application Service (MTAS) application form was heavily criticized for being vague with ‘soft’ questions and there were serious breaches of security of the MTAS website.[26] As a result, MTAS was abandoned for 2008 recruitment in favor of local applications at a deanery level and there were no limits to the number of deaneries a junior doctor could apply to; neither were there any limits to the number of different specialties a doctor could apply to as long as they stay to a level in accordance with their experience and competencies gained to date against a specific person specification. The 2009 psychiatric recruitment saw the Royal College coordinating nationalized application and we are yet to analyse formally the merits of this approach although the early signs are that it has been very successful. The foundation program itself is also currently under review and there has been speculation in the medical press of a possible shift towards a F1 year followed by a three-year core training program followed by further specialist training although it now seems as if this will not change in the foreseeable future.

These limitations in the new structure have certainly posed some practical difficulties and it has been a stormy transition for the trainees caught in the middle of this system overhaul. It is interesting to note that some of the original aims and structures of these plans still remain controversial and medical professionals appear divided on many of these initiatives. The major problem was observed in the introduction of this new system and failure of the MTAS through the online application for postgraduate medical recruitment in 2007. There is however a positive note that despite the chaotic introduction of the new system and initial difficulties in its implementation, there is a skepticism that these changes will have long-lasting effects in the current postgraduate medical training system. There is still a lot more work to be done on the new assessment tools and it is hoped that we will get more experience for their reliability and validity in the coming years.
